# *Ilex decapetala* (Aquifoliaceae), a new holly species from Vietnam revealed by molecular and morphological approaches

**DOI:** 10.3897/phytokeys.278.197441

**Published:** 2026-08-03

**Authors:** Yi Yang, Wan Hu, Tao Huang, Jing Wang, Khang Sinh Nguyen, Ninh Khac Ban, Van Thanh Bui, Le Xuan Dac, Maxim S. Nuraliev

**Affiliations:** 1 Jiangxi Provincial Key Laboratory of Improved Variety Breeding and Efficient Utilization of Native Tree Species, Forestry College, Jiangxi Agricultural University, Nanchang 330045, China Jiangxi Provincial Key Laboratory of Improved Variety Breeding and Efficient Utilization of Native Tree Species, Forestry College, Jiangxi Agricultural University Nanchang China; 2 Lushan Botanical Garden, Jiangxi Province and Chinese Academy of Sciences, Jiujiang 332900, China Joint Vietnam-Russia Tropical Science and Technology Research Center Hanoi Vietnam; 3 Institute of Biology, Vietnam Academy of Science and Technology, Hanoi 10072, Vietnam Department of Higher Plants, Biological Faculty, M. V. Lomonosov Moscow State University Moscow Russia; 4 Institute of Chemistry, Vietnam Academy of Science and Technology, Hanoi 10072, Vietnam Institute of Biology, Vietnam Academy of Science and Technology Hanoi Vietnam; 5 Graduate University of Science and Technology, Vietnam Academy of Science and Technology, Hanoi 10072, Vietnam Institute of Chemistry, Vietnam Academy of Science and Technology Hanoi Vietnam; 6 Joint Vietnam-Russia Tropical Science and Technology Research Center, Hanoi 122000, Vietnam Lushan Botanical Garden, Jiangxi Province and Chinese Academy of Sciences Jiujiang China; 7 Joint Vietnam-Russia Tropical Science and Technology Research Center, A. N. Severtsov Institute of Ecology and Evolution, Russian Academy of Sciences, Moscow 119071, Russia Graduate University of Science and Technology, Vietnam Academy of Science and Technology Hanoi Vietnam; 8 Department of Higher Plants, Biological Faculty, M. V. Lomonosov Moscow State University, Moscow 119234, Russia Joint Vietnam-Russia Tropical Science and Technology Research Center, A. N. Severtsov Institute of Ecology and Evolution, Russian Academy of Sciences Moscow Russia

**Keywords:** Floral merism, Indo-Burma, Kon Tum, phylogeny, Quang Nam, Thua Thien Hue

## Abstract

A new species, *Ilex decapetala*, is described and illustrated from central Vietnam. It is distinguished by its female flowers with (8–)9–10(–11) pink petals (mostly 10); such a high floral merism is rare within the genus and, on top of this rarity, no other known species of *Ilex* combines such a high merism with pink corolla. Phylogenetic analyses, based on three nuclear markers (ITS, ETS and *nep*GS), place the new species in *Ilex* sect. *Prinifoliae* with strong support, where it is sister to a clade comprising *I. angulata*, *I. hainanensis*, *I. pubescens* and *I. stewardii*. Morphologically, *I. decapetala* resembles *I. stewardii* most, but differs in having more numerous flowers in female inflorescences (3–18 vs. 1–5) and a higher merism of corolla and androecium of female flowers (8–11 vs. 6–7). In addition, the new species shows similarity to *I. umbellulata*, which also occurs in central Vietnam, but the latter is distinguished by white flowers with 4–5 petals and staminodes.

## Introduction

*Ilex* L. is the sole genus within the family Aquifoliaceae; it comprises the plants commonly known as hollies (Powell et al. 2000; Manen et al. 2010). It consists of 571 recognised species and several (ca. 7) nothospecies of dioecious trees and shrubs (POWO, https://powo.science.kew.org/; Loizeau et al. 2005; Liu et al. 2024). The distinguishing features of *Ilex* include the presence of small stipules, cymose or simplified-cymose (raceme-like, panicle-like or single-flowered) inflorescences, gynoecium usually lacking styles, capitate stigmas and drupes with 4–6(–23) pyrenes (Loizeau et al. 2016). Economically, the holly genus is important for its widespread usage as the sources of beverages [e.g. kudingcha (*I. kaushue* S.Y.Hu and *I. latifolia* Thunb.), yaupon (*I. vomitoria* Aiton) and yerba mate (*I. paraguariensis* A.St.-Hil.)], garden ornamentals [e.g. American holly (*I. opaca* Aiton), Chinese holly (*I. cornuta* Lindl. & Paxton) and English holly (*I. aquifolium* L.)] and traditional medicines [e.g. gangmei (*I. asprella* Champ. ex Benth.) and jiubiying (*I. rotunda* Thunb.)] (Galle 1997; Hao et al. 2013; Gan et al. 2018; Yao et al. 2022; Zeng et al. 2022; Wang et al. 2025).

Like most species-rich genera, the classification of *Ilex* has undergone numerous revisions. Both Loesener (1942) and Galle (1997) grouped the holly species into several (five and three, respectively) subgenera, which were further subdivided into sections and series. However, neither Loesener’s nor Galle’s treatments are consistent with the molecular phylogenetic reconstructions (Cuénoud et al. 2000; Powell et al. 2000; Manen et al. 2002, 2010; Gottlieb et al. 2005; Selbach-Schnadelbach et al. 2009; Yang et al. 2023; Xu et al. 2025). Yang et al. (2023) proposed a comprehensive classification of *Ilex* that takes into account the phylogenetic data, morphological traits and distribution patterns. In this new classification, 14 sections of the genus were recognised, whereas its subdivision into subgenera was not used.

*Ilex* has a sub-cosmopolitan natural distribution range, with its centres of species diversity in the subtropical and tropical regions of East and Southeast Asia and in Central and South America (Andrews 2002; Loizeau et al. 2005; Yang et al. 2023). Twenty-six holly species and one variety were described as new to science in the past decade as a result of the recent large-scale field surveys (Jiang et al. 2017, 2020; Pruesapan et al. 2017; Xu et al. 2017; Yang et al. 2018; Grande Allende 2019; He 2019; Lozada-Pérez 2019; Tagane et al. 2020; Ford et al. 2021; Pruesapan and van Welzen 2021, 2025; Rodríguez 2022; Ying 2022, 2023; Barriera 2023; Chen et al. 2023; Lin et al. 2023; González-Murillo and Zamudio 2024; Dang et al. 2025; Zhao et al. 2026). Notably, 19 of these new species were found in southern China and the Indochina Peninsula, i.e. mostly within the Indo-Burma biodiversity hotspot as defined by Myers et al. (2000) and Brooks et al. (2006). The continuing findings indicate that the inventory of the species diversity of *Ilex* is far from being complete.

As part of the Indo-Burma region, Vietnam holds a rich diversity of vascular plants (ca. 12,000 or over 20,000 species, as estimated by Nguyen (2001–2005); Pham (2003); Regalado Jr et al. (2005); Luu-dam et al. (2023)). With respect to *Ilex*, 41 its species have been documented in Vietnam to date (Nguyen 2001–2005; Bui et al. 2020; Tagane et al. 2020).

Here, we describe a further new species of *Ilex*, discovered in Vietnam during the field investigations of the Vietnam-Russia Tropical Center conducted in 2015 and 2019 as well as during those of the Vietnam Academy of Science and Technology in 2026. In support of our taxonomic conclusions, we also provide the results of phylogenetic reconstructions, based on three nuclear markers.

## Materials and methods

### Phylogenetic analyses

The choice of phylogenetic markers follows Yang et al. (2023). Three nuclear DNA loci were used, i.e. the ITS region (including the internal transcribed spacers 1 and 2 and the 5.8S rRNA gene), the external transcribed spacer (ETS) and the protein-coding *nep*GS gene. In total, 203 samples representing 190 *Ilex* species were included in the analyses; 13 samples were newly sequenced, whereas the other accessions were obtained from Yang et al. (2023). The genera *Helwingia* Willd. (Helwingiaceae) and *Phyllonoma* Willd. (Phyllonomaceae) were selected as outgroups, based on recent phylogenetic studies (Zhang et al. 2020; Li et al. 2021). Details of DNA extraction, primers, PCR amplification, sequencing and sequence alignment follow Jiang et al. (2017) and Yang et al. (2018, 2023). Aligned DNA sequences are available in Suppl. material 1. Voucher and GenBank accession information are shown in Suppl. material 2.

Phylogenetic reconstructions were performed using Bayesian Inference (BI) and Maximum-Likelihood (ML) approaches, based on the concatenated dataset (ITS + ETS + *nep*GS, 2370 base pairs in length). The optimal nucleotide substitution model for the ML analysis, TPM3u+I+R3, was found by ModelFinder (Kalyaanamoorthy et al. 2017) using the Bayesian information criterion. ML analysis was performed in IQ-TREE v.3.0.1 (Minh et al. 2020) with 1,000 ultrafast bootstrap replicates (Hoang et al. 2018). BI analysis was implemented in MrBayes v.3.2.7 (Ronquist et al. 2012) using a mixed substitution model with invariant sites and gamma-distributed rate variation (nst = mixed, rates = invgamma), with running 10,000,000 generations, sampling every 1,000 generations and discarding the first 25% of the trees as burn-in.

### Morphological assessment

Related literature, especially the protologues of all published names of holly species found in Asia, was reviewed and collated. To achieve a precise description of the new species, we adopted the diagnostic characters summarised by Hu (1949) and Yang et al. (2023). Morphological description of the new species was derived from an assessment of the herbarium specimens and corresponding field photographs. Digital specimen images of several other holly species housed in IBK, KUN, P and PE were also checked via the websites of Chinese Virtual Herbarium (CVH, https://www.cvh.ac.cn/) and Global Biodiversity Information Facility (GBIF, https://www.gbif.org/). To ensure consideration of all the available data on morphology and geographical distribution of the new species, all digitised *Ilex* specimens from Vietnam accessible through the GBIF database and in the HN online collections were examined. In addition, targeted search for individuals of the new species and its key plant parts (including male and female flowers and fruits) was conducted in the three localities reported below as well as in several other protected areas of central Vietnam. The conservation status was assessed following the IUCN Red List Categories and Criteria (IUCN Standards and Petitions Committee 2024). Herbarium acronyms follow Index Herbariorum (https://sweetgum.nybg.org/science/ih/).

## Results

### Phylogenetic placement

**Figure 1.**
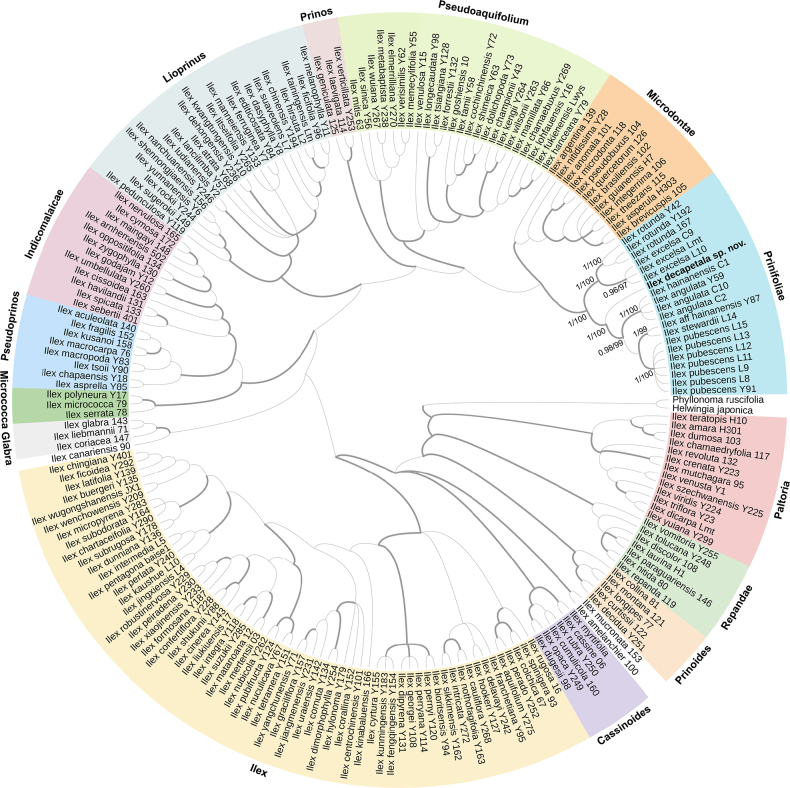
Bayesian consensus tree (50% majority‐rule consensus) for concatenated dataset (ITS + ETS + *nep*GS), with 14 sections annotated. Nodes with both BI-PP > 0.95 and ML-BP > 85 are shown in bold.

The BI and ML trees (Fig. 1, see also the Suppl. materials 3, 4) generated in this study show minimal topological conflict, but the BI tree has higher support of several branches. Both are congruent with the reconstructions by Yang et al. (2023), supporting the monophyly of *Ilex* and its subdivision into 15 major clades. The new species is placed within the clade corresponding to *Ilex* sect. *Prinifoliae* Loes. (Fig. 1), which is strongly supported (BI-PP/ML-BP: 1/100). This clade consists of seven species. It is subdivided into two subclades (1/100 for each). The new species is the basalmost in one of the subclades, being sister to a clade (0.98/99) that includes the subclade (0.71/98) *I. pubescens* Hook. & Arn. (1/100) + (*I. stewardii* S.Y.Hu + *I. aff. hainanensis* (1/99)) and a subclade (1/100) containing *I. angulata* Merr. & Chun and *I. hainanensis* Merr. The second subclade of *Ilex* sect. *Prinifoliae* comprises the clades of *I. excelsa* (Wall.) Hook.f. (0.96/97) and *I. rotunda* Thunb. (1/100).

### Taxonomic treatment

#### Ilex decapetala

Taxon classificationPlantaeFagalesFagaceae

Yi Yang, Nuraliev & W.Hu sp. nov.

E5FFAE7E-8BA0-5DC7-8727-FB70EB9CB6FB

urn:lsid:ipni.org:names:77393586-1

Figs 2, 3, 4

##### Type.

Vietnam • Quang Nam: Nam Giang District, Song Thanh Nature Reserve, forest, river bank, elev. 740 m a.s.l., 15°33'14"N, 107°24'24"E, 11 May 2019, *M.S. Nuraliev 2540* [(holotype MW! (MW0756601, image available at https://www.gbif.org/occurrence/3004105330 and https://plant.depo.msu.ru/open/public/item/MW0756601); isotypes LBG! (LBG00161813), MW! (MW0756602, https://www.gbif.org/occurrence/3004115330 and https://plant.depo.msu.ru/open/public/item/MW0756602)].

**Figure 2.**
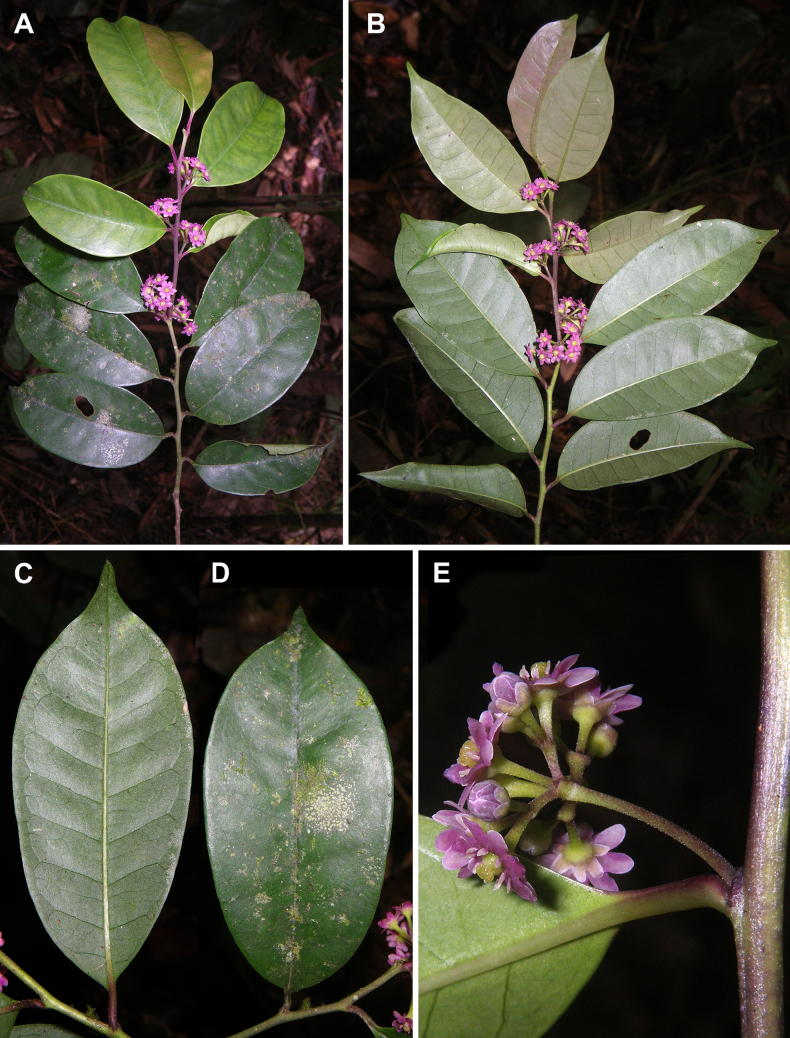
*Ilex decapetala*, female plants. **A, B**. Flowering branch, view from above and from below; **C, D**. Leaf, abaxial and adaxial side; **E**. Inflorescence in leaf axil, side view (note the stipule). *Nuraliev 2540* (**A–D**); *Nuraliev 1411* (**E**). Photos by M.S. Nuraliev.

**Figure 3.**
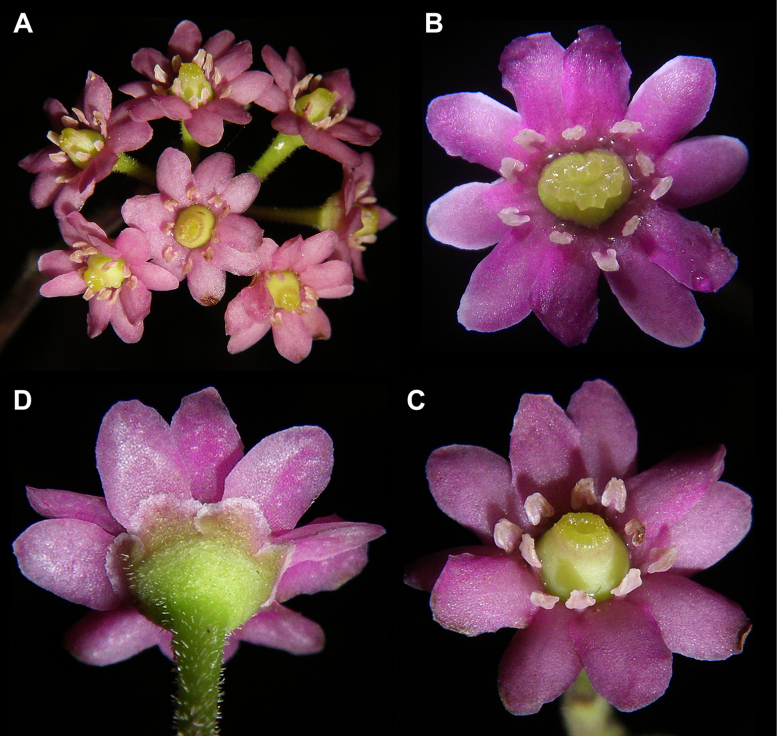
*Ilex decapetala*, female plants. **A**. Inflorescence, front view; **B, C**. Flower, top and semi-side view; **D**. Flower, view from below. *Nuraliev 2540* (**A, C, D**); *Nuraliev 1411* (**B**). Photos by M.S. Nuraliev.

**Figure 4.**
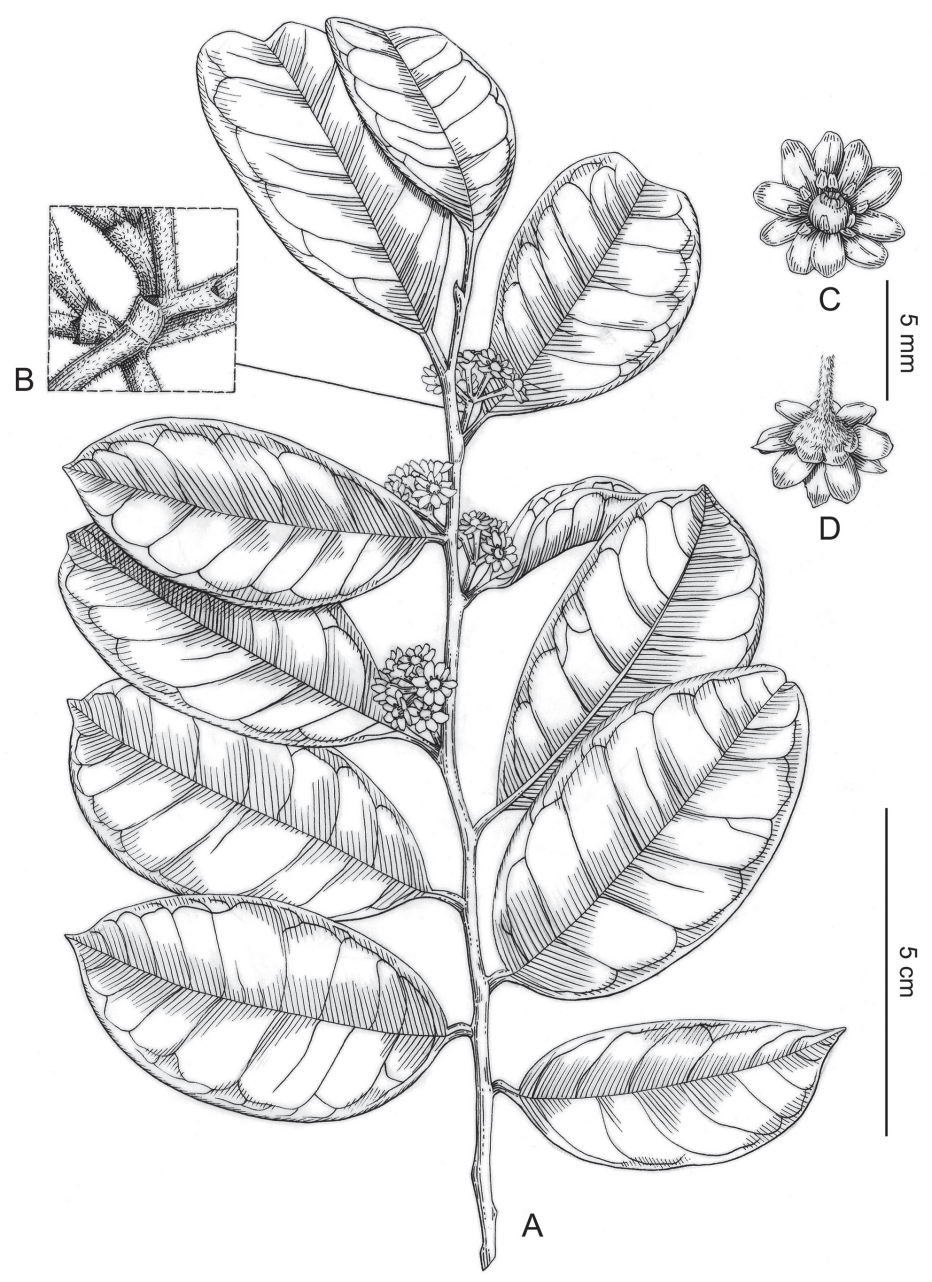
*Ilex decapetala*, female individual. **A**. Flowering branch; **B**. Portion of inflorescence, showing peduncle, branches and bracts; **C**. Flower, top view; **D**. Flower, view from below. Drawn from an image of the holotype (*Nuraliev 2540*, MW0756601) by Y. Chen.

##### Description.

***Small trees or shrubs*** about 4 m tall, most likely evergreen. ***Branches*** of the current year purplish or brown-purple, puberulent; second-year branches green, glabrescent; third-year and older branches greyish to brownish-grey, glabrescent. ***Petioles*** 6–10 mm long, green to purplish, puberulent, but glabrescent with age. ***Leaf blades*** lanceolate, elliptic, ovate-elliptic or obovate-elliptic, 3.7–8.5 cm long, 1.8–3.4 cm wide, chartaceous, both surfaces glabrous, sometimes sparsely puberulent along the mid-vein abaxially, secondary veins 7–11 pairs, base cuneate to obtuse, margin entire, apex acuminate to long acuminate. ***Stipules*** tiny, triangular to broadly triangular, dark brown, glabrous, base rounded, margin entire, apex acute, caducous. ***Male inflorescences*** and flowers unknown. ***Female inflorescences*** axillary, occurring on current-year and/or second-year branches, cymose, corymbiform, ca. 1.5–2 cm in diameter, with (1–)3–18 flowers per inflorescence (rarely a branch with several cymes resembling a thyrse due to development of bracts instead of foliage leaves); peduncles 5–14 mm long, puberulent; bracts tiny, triangular to broadly triangular, brown to dark brown; rachises of thyrse-like branches 2–4 cm long, puberulent. ***Female flowers***: pedicel 4–9 mm long, puberulent; calyx ca. 2.5 mm in diameter, yellowish-green and scarious along margin, puberulent outside and glabrous inside, 6- or 7-lobed, lobes broadly deltoid and overlapping each other; corolla 4.5–6 mm in diameter when fully opened, tube ca. 1 mm long, lobes (8–)9–10(–11), elliptic, 1.8–2.1 mm long, 0.8–1 mm wide, pink to pink-violet, glabrous, rarely with sparse hairs along margin, apex rounded; staminodes 9–10(–11), usually in the same number as the petals, ca. 2/5 as long as the petals, glabrous, filaments pink, sterile anthers deltoid, pinkish-white; ovary superior, ca. 1.5 mm in height, subglobose or elliptic in top view due to the carpels arranged along a line, green, glabrous, with 9–10 locules, each locule 1-ovuled; stigma elliptic-discoid, green, 9–10-lobed. ***Fruits*** when young green, glabrous, with persistent puberulent calyx; ripe fruits unknown.

##### Etymology.

The species is named for its female flowers that mostly have 10 petals, which makes it distinct within the holly genus, because the flowers of *Ilex* are predominantly 4–7-merous.

##### Phenology.

*Ilex decapetala* starts to flower in April or May; fruiting season is unknown.

##### Distribution.

**Figure 5.**
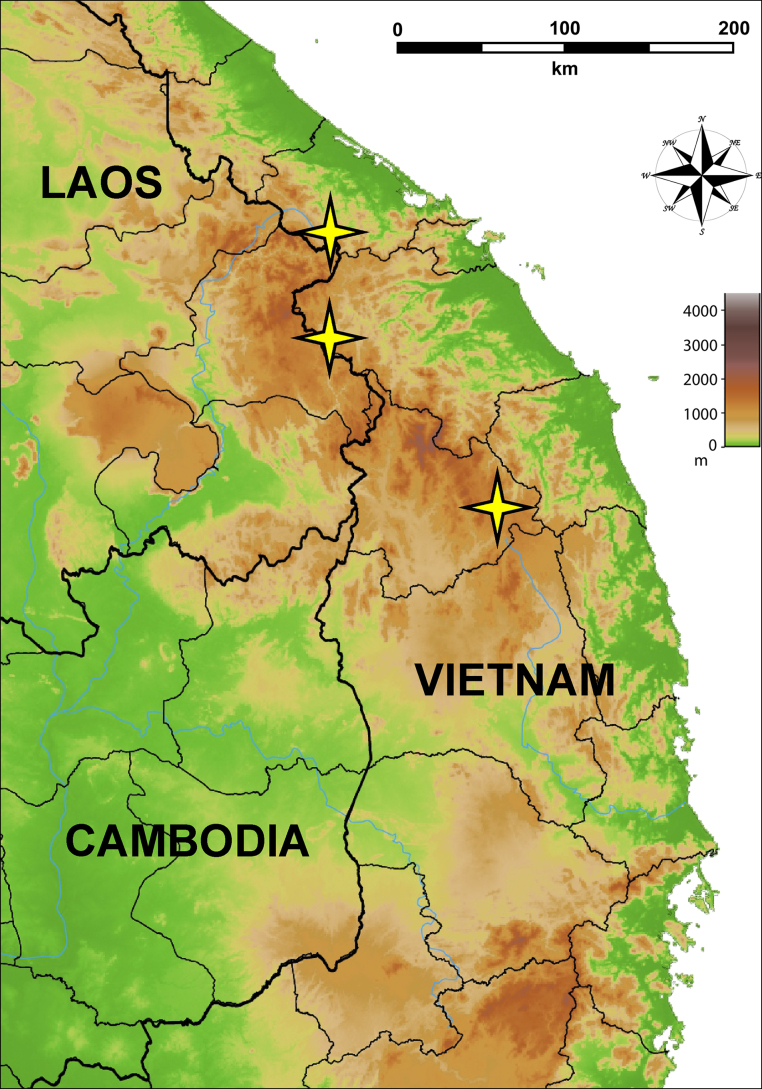
Distribution of *Ilex decapetala* (the map is not aimed to show countries in their entire borders). Currently known distribution sites of the new species are indicated by yellow stars.

*Ilex decapetala* is currently known from three neighbouring provinces of central Vietnam, i.e. Kon Tum, Quang Nam and Thua Thien Hue (Fig. 5). Two of the localities are within several km of the border with Laos, which means that the presence of the species in Laos is highly possible.

##### Preliminary conservation assessment.

DD (Data Deficient). Three populations were recorded, with the two most distant ones located 185 km apart from each other. Further field observations on the species and an assessment of the quality of its habitats are needed.

##### Paratypes.

Vietnam • Thua Thien Hue: A Luoi 4 Commune, A Roang 2 Village, remnants of primary evergreen broad-leaved forest on sandstone mountain, around point 16.145186°N, 107.416529°E, elev. 600–700 m a.s.l., 27 April 2026, *Ninh Khac Ban, Bui Van Thanh, Khang Sinh Nguyen et al. AL 603* (HN!); • ibid., 10 June 2026, *Ninh Khac Ban et al. AL 817* (HN!); • Kon Tum: Kon Plong District, Mang Canh Municipality, Thach Nham protected forest, 13 km NNE from Mang Den town, in the forest, river bank, elev. 1050 m a.s.l., 14°42'35"N, 108°19'00"E, 22 April 2015, *M.S. Nuraliev 1411* [MW! (MW0750769, image available at https://www.gbif.org/occurrence/1697868135 and https://plant.depo.msu.ru/open/public/item/MW0750769)].

## Discussion

In our phylogenetic reconstructions, *Ilex decapetala* is firmly placed within *Ilex* sect. *Prinifoliae*. This placement is corroborated by morphological data: the entire leaves, cymose female inflorescences and pink petals of the new species are consistent with the characteristics of *Ilex* sect. *Prinifoliae* (Yang et al. 2023). Morphologically, *I. decapetala* bears the closest resemblance to *I. stewardii* (another member of *Ilex* sect. *Prinifoliae*), sharing numerous vegetative and floral traits, including branchlet colour, shape, size, texture and number of secondary veins of the leaf blade, calyx merism and petal colour. However, *I. decapetala* is readily distinguished from *I. stewardii* by its female inflorescences bearing (1–)3–18 (vs. 1–5) flowers, as well as female flowers possessing a higher merism of corolla and androecium [(8–)9–10(–11) vs. 6–7]. In addition, *I. decapetala* exhibits a degree of morphological affinity with *I. umbellulata* (Wall.) Loes. [erroneously referred to by Pham (2003) as “*I. umbellata*”], a member of *Ilex* sect. *Indicomalaicae* Loes. (see Fig. 1) that also occurs in Vietnam, in particular by sharing entire leaves and cymose female inflorescences with corymb-like shape. Nonetheless, *I. umbellulata* is clearly differentiated by its female flowers with white corolla and markedly fewer petals (4–5) and staminodes (4–5). A comprehensive comparison of these three species is presented in Table 1. More broadly, *I. decapetala* appears, to the best of our knowledge (Loesener 1901; Galle 1997; Andrews 1998; Yang et al. 2023), unique within the genus in having a combination of pink corolla and the merism of corolla and androecium higher than eight.

**Table 1.** A morphological comparison of *Ilex decapetala*, *I. stewardii* and *I. umbellulata*. The relevant characters of *I. stewardii* and *I. umbellulata* are referenced from Chen et al. (2008).

		***I. decapetala***	***I. stewardii***	***I. umbellulata***
Branchlets	colour	purplish or purple	green, purplish or purple	green
	indumentum	puberulent	sparsely puberulent	glabrous
Petiole	length	6–10 mm	5–8 mm	10–12 mm
	indumentum	puberulent, but glabrescent with age	puberulent	glabrous
Leaf blade	size	3.7–8.5 × 1.8–3.4 cm	5–8 × 1.5–3 cm	7–15 × (3.5–)5–6 cm
	shape of apex	acuminate to long acuminate	long acuminate	abruptly acuminate or obtuse
Female inflorescence	number of flowers	(1–)3–18	1–5	3–15
	peduncle length	5–14 mm	3–12 mm	10–20 mm
Female flower	merism of corolla and androecium	(8–)9–10(–11)	6–7	4–5
	petal colour	pink to pink-violet	pink to pink-violet	white

Although the combination of the obtained morphological data (especially the characters of the female flowers) and the phylogenetic results unequivocally indicates the identity of the studied specimens as a species new to science, the gross morphology of *Ilex decapetala* remains incompletely known. In particular, we highlight the absence of any information on its male flowers and mature fruits (including pyrenes); further investigations are required to fill these gaps.

The occurrence of flowers with more than eight petals and staminodes, as observed in *Ilex decapetala*, is a notable phenomenon within *Ilex*, whose flowers are predominantly 4–7-merous (Galle 1997; Chen et al. 2008; Loizeau et al. 2016). While uncommon, this trait is not unique to this species. High floral merism has been documented in several other hollies, for example, *I. fragilis* Hook.f. (6–13 petals and staminodes) in sect. *Pseudoprinos* S.Y. Hu, *I. anomala* Hook. & Arn. (10–18) in sect. *Microdontae* Loes. and *I. arnhemensis* (F.Muell.) Loes. (12–13) and *I. zygophylla* Merr. (12) in sect. *Indicomalaicae* (Yang et al. 2023). The merism of the male flowers of *Ilex* is known to be equal or lower than in the female flowers. The scattered distribution of the species with high floral merism across different sections of *Ilex* strongly suggests that this is a derived trait which has evolved multiple times independently within the holly genus.

## Supplementary Material

XML Treatment for Ilex decapetala

## Additional information

### Artificial Intelligence (AI) use

The authors accept full responsibility for the content of the manuscript, including the disclosure of any use of AI.

No AI tools were used in the preparation of this manuscript.

### Author ORCIDs

Y. Yang https://orcid.org/0000-0003-1996-5446

W. Hu https://orcid.org/0009-0003-7282-8333

T. Huang https://orcid.org/0009-0001-7340-1652

J. Wang https://orcid.org/0009-0003-7198-8191

K. Sinh Nguyen https://orcid.org/0000-0001-5171-4140

N.K. Ban https://orcid.org/0000-0002-1249-1500

V.T. Bui https://orcid.org/0000-0002-0128-1196

L. Xuan Dac https://orcid.org/0000-0003-4586-6214

M.S. Nuraliev https://orcid.org/0000-0001-8291-2633

### Data availability

All of the data that support the findings of this study are available in the main text or Supplementary Information.

## Supplementary materials

Supplementary material 1 Aligned DNA sequences Data type fas Explanation note Aligned sequences used for phylogenetic analyses in this study. https://binary.pensoft.net/file/1736297This dataset is made available under the Open Database License (http://opendatacommons.org/licenses/odbl/1.0/). The Open Database License (ODbL) is a license agreement intended to allow users to freely share, modify, and use this Dataset while maintaining this same freedom for others, provided that the original source and author(s) are credited. Yi Yang, Wan Hu, Tao Huang, Jing Wang, Khang Sinh Nguyen, Ninh Khac Ban, Van Thanh Bui, Le Xuan Dac, Maxim S. Nuraliev

Supplementary material 2 Voucher and GenBank accession information Data type xlsx Explanation note Voucher and GenBank accession of the samples/ sequences used for phylogenetic analyses in this study. https://binary.pensoft.net/file/1736298This dataset is made available under the Open Database License (http://opendatacommons.org/licenses/odbl/1.0/). The Open Database License (ODbL) is a license agreement intended to allow users to freely share, modify, and use this Dataset while maintaining this same freedom for others, provided that the original source and author(s) are credited. Yi Yang, Wan Hu, Tao Huang, Jing Wang, Khang Sinh Nguyen, Ninh Khac Ban, Van Thanh Bui, Le Xuan Dac, Maxim S. Nuraliev

Supplementary material 3 BI tree Data type pdf Explanation note Bayesian consensus tree (50% majority‐rule consensus) for concatenated dataset (ITS + ETS + nepGS). https://binary.pensoft.net/file/1736299This dataset is made available under the Open Database License (http://opendatacommons.org/licenses/odbl/1.0/). The Open Database License (ODbL) is a license agreement intended to allow users to freely share, modify, and use this Dataset while maintaining this same freedom for others, provided that the original source and author(s) are credited. Yi Yang, Wan Hu, Tao Huang, Jing Wang, Khang Sinh Nguyen, Ninh Khac Ban, Van Thanh Bui, Le Xuan Dac, Maxim S. Nuraliev

Supplementary material 4 ML tree Data type pdf Explanation note Maximum likelihood tree for concatenated dataset (ITS + ETS + nepGS). https://binary.pensoft.net/file/1736300This dataset is made available under the Open Database License (http://opendatacommons.org/licenses/odbl/1.0/). The Open Database License (ODbL) is a license agreement intended to allow users to freely share, modify, and use this Dataset while maintaining this same freedom for others, provided that the original source and author(s) are credited. Yi Yang, Wan Hu, Tao Huang, Jing Wang, Khang Sinh Nguyen, Ninh Khac Ban, Van Thanh Bui, Le Xuan Dac, Maxim S. Nuraliev
