# Supplementary material for: Ilex decapetala (Aquifoliaceae), a new holly species from Vietnam revealed by molecular and morphological approaches
Source: PhytoKeys. 2026 Aug 3;278:75–90. doi: 10.3897/phytokeys.278.197441 (PMC13458434; doi:10.3897/phytokeys.278.197441)
Supplement: Supplementary file 1 [file phytokeys-278-075_article-197441__-s003.pdf]

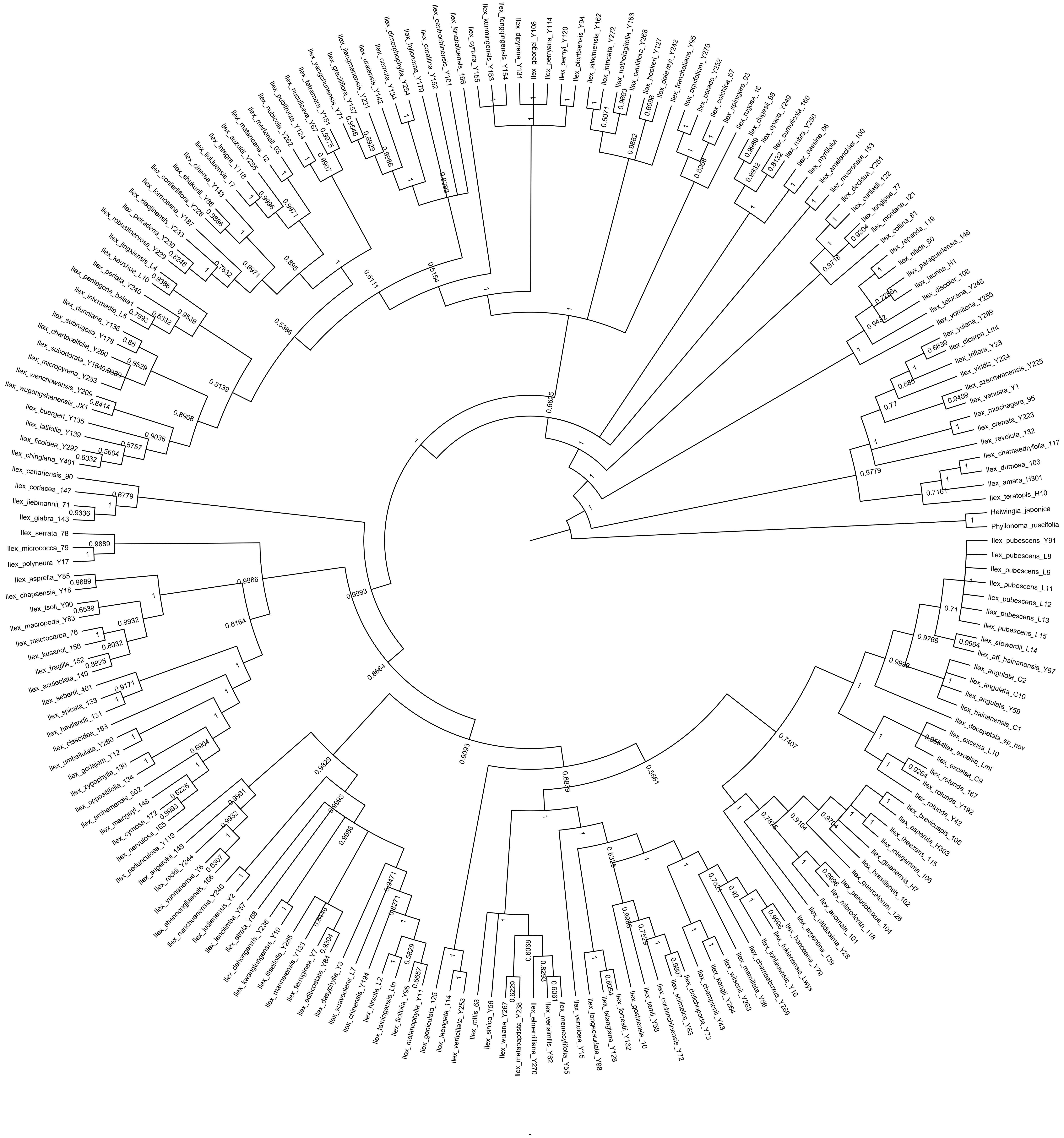
Helwingia_japonica
Phyllonoma_ruscifolia
Ilex_pubescens_Y91
Ilex_pubescens_L8
Ilex_pubescens_L9
Ilex_pubescens_L11
Ilex_pubescens_L12
Ilex_pubescens_L13
Ilex_pubescens_L15
Ilex_stewardii_L14
Ilex_aff_hainanensis_Y87
Ilex_angulata_C2
Ilex_angulata_C10
Ilex_angulata_Y59
Ilex_hainanensis_C1
Ilex_decapetala_sp_nov
Ilex_revoluta_132
Ilex_chamaedryfolia_117
Ilex_dumosa_103
Ilex_amara_H301
Ilex_teratopis_H10
Ilex_crenata_Y223
Ilex_mutchagara_95
Ilex_venusta_Y1
Ilex_szechwanensis_Y225
Ilex_canariensis_90
Ilex_coriacea_147
Ilex_liebmannii_71
Ilex_glabra_143
Ilex_serrata_78
Ilex_micrococca_79
Ilex_polyneura_Y17
Ilex_asprella_Y85
Ilex_chapaensis_Y18
Ilex_tsoii_Y90
Ilex_macropoda_Y83
Ilex_macrocarpa_76
Ilex_kusanoi_158
Ilex_fragilis_152
Ilex_aculeolata_140
Ilex_buergeri_Y135
Ilex_latifolia_Y139
Ilex_ficoidea_Y292
Ilex_chingiana_Y401
0.07
