# Supplementary material for: Ilex decapetala (Aquifoliaceae), a new holly species from Vietnam revealed by molecular and morphological approaches
Source: PhytoKeys. 2026 Aug 3;278:75–90. doi: 10.3897/phytokeys.278.197441 (PMC13458434; doi:10.3897/phytokeys.278.197441)
Supplement: Supplementary file 2 [file phytokeys-278-075_article-197441__-s004.pdf]

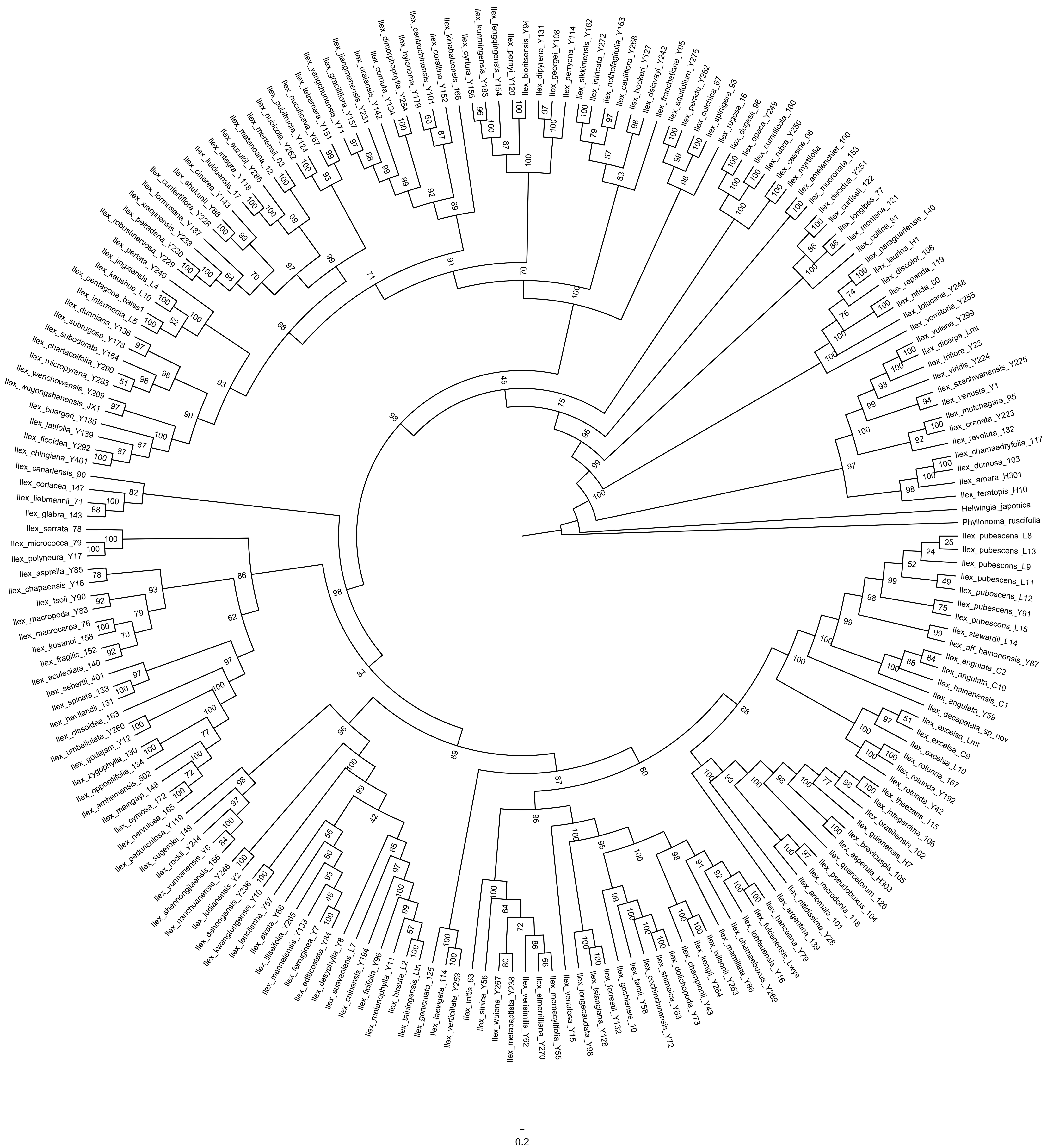

0.2
